# Jejunal mucosa proteomics unravel metabolic adaptive processes to mild chronic heat stress in dairy cows

**DOI:** 10.1038/s41598-021-92053-x

**Published:** 2021-06-14

**Authors:** Franziska Koch, Dirk Albrecht, Solvig Görs, Björn Kuhla

**Affiliations:** 1 Institute of Nutritional Physiology “Oskar Kellner”, Leibniz Institute for Farm Animal Biology (FBN), Wilhelm-Stahl-Allee 2, 18196 Dummerstorf, Germany; 2 Institute of Microbiology, Ernst-Moritz-Arndt-University, Felix-Hausdorff-Straße 8, 17487 Greifswald, Germany

**Keywords:** Molecular biology, Physiology

## Abstract

Climate change affects the duration and intensity of heat waves during summer months and jeopardizes animal health and welfare. High ambient temperatures cause heat stress in dairy cows resulting in a reduction of milk yield, feed intake, and alterations in gut barrier function. The objectives of this study were to investigate the mucosal amino acid, glucose and lactate metabolism, as well as the proteomic response of the small intestine in heat stressed (HS) Holstein dairy cows. Cows of the HS group (n = 5) were exposed for 4 days to 28 °C (THI = 76) in a climate chamber. Percentage decrease in daily ad libitum intake of HS cows was calculated to provide isocaloric energy intake to pair-fed control cows kept at 15 °C (THI = 60) for 4 days. The metabolite, mRNA and proteomic analyses revealed that HS induced incorrect protein folding, cellular destabilization, increased proteolytic degradation and protein kinase inhibitor activity, reduced glycolysis, and activation of NF-κB signaling, uronate cycling, pentose phosphate pathway, fatty acid and amino acid catabolism, mitochondrial respiration, ATPase activity and the antioxidative defence system. Our results highlight adaptive metabolic and immune mechanisms attempting to maintain the biological function in the small intestine of heat-stressed dairy cows.

## Introduction

By the end of the twenty-first century, mean ambient temperatures are predicted to increase resulting in a greater climate warming in the Northern hemisphere^1^. Consequently, both humans and animals are exposed to heat waves during summer months at a higher frequency, intensity and duration affecting behavior, health and welfare^2,3^. Under higher ambient temperature and humidity, specifically heat intolerant species, e.g. dairy cows, lose their ability to dissipate heat, causing an increase of animal´s body temperature and heat stress^4^. The thermoneutral zone of a dairy cow depends on her breed, individual body size, anatomic appearance and milk yield. Several studies indicated that Jersey cows are less sensitive to high ambient temperatures than Brown Swiss or Holstein cows, whose thermoneutral zone ranges between 5–20 °C and is below a temperature-humidity-index (THI) of 68^5–7^. When the temperature-humidity-index (THI) increases from 60–64 to 73–92, feed intake is reduced by 35–40% in dairy cows^8–10^, thus impairing the nutrient and energy supply for the organism. The accompanied heat stress adversely affects a variety of vital parameters, e.g. respiration and heart rate^8,10^, body temperature^9^ and drinking and feeding behavior^8,11^.

The gastrointestinal tract plays an important role in the absorption and digestion of nutrients and forms a tight barrier between the external and internal environment. The small intestine is particularly responsive to hyperthermia^12^. High ambient temperatures promote the blood circulation of the skin in order to dissipate heat, but the decrease in visceral blood flow induces hypoxic episodes with impaired gut barrier function and bacterial translocation^13,14^. Electron microscopic studies of different parts of the intestine report the most severe damages of the mucosal epithelia with desquamification of the villi in the jejunum of heat-stressed rats^15^ and pigs^16^.

The intestinal mucosa is a multicellular tissue predominantly consisting of enterocytes and lymphoid cells, which all have different preferences for nutrient fuels^17^. Glutamine, glucose and ketones, and to a very little extent short-chain fatty acids, serve as major energy fuels for enterocytes^18^. Lymphocytes utilize glutamine and glucose as major energy sources^17^. During intestinal injuries, the enterocyte’s glutamine and glucose catabolism is reduced and a switch from energy-efficient oxidative phosphorylation to less efficient anerobic glycolysis was observed^17^. This metabolic shift compromises the enterocyte’s metabolism to spare energy fuels for the activation of lymphocytes and macrophages^17^. First data of heat-stressed dairy cows revealed immune cells of myeloid origin in the *Muscularis mucosae* in mid-jejunum indicating a potential activation of an immune response against bacterial translocation to maintain gut barrier function^14^. Such a low-grade local inflammation causes significant energetic costs, which has to be met by fuels originating from the circulation or from non-immune cells of the intestine, i.e. mucosal cells. Whether changes in the glucose and glutamine metabolism or other metabolic pathways of the intestine contribute to facilitate energetically the local immune response during heat stress is not known.

Heat shock proteins (HSPs) play a major role in stabilizing enzymes to maintain enzyme activity under high ambient temperatures and to avoid protein misfolding and malfunction^19^. The accumulation of denaturated proteins leads to the loss of cell viability under heat stress^19^. Recent studies have shown an increase of HSPs in serum of beef calves kept at high versus low THI^20^ and in liver of dairy cows after exposure to high ambient temperature for 9 days relative to pair-fed controls at thermoneutrality^21^. The abundance of HSP70, HSP60 and HSP47 also increased in avian jejunum after 6 h of heat stress^22^. Thus, alterations in the expression of HSPs and their chaperone function seems to be activated already within hours after induction of heat stress and maintained over several days during high ambient temperatures.

Here, we hypothesized that mild chronic heat stress in dairy cows shifts glucose towards glutamine and lactate metabolism likely to preserve glucose for immune responses, and that heat stress alters the expression of HSP and many further proteins to maintain the biological function of the challenged jejunal mucosa. To examine these hypotheses, mid-lactating Holstein dairy cows were either subjected to heat stress or pair-feeding at thermoneutrality for 4 days before sampling for jejunal mucosa. Tissue samples were analysed for mucosal amino acid, glucose and lactate concentrations, the activity of enzymes involved in their degradation, as well as by an untargeted proteomics approach. We further examined if expression differences of distinct proteins could be also be found at mRNA level.

## Results

### Temperature, humidity, THI, feed intake and milk yield

**Figure 1:**
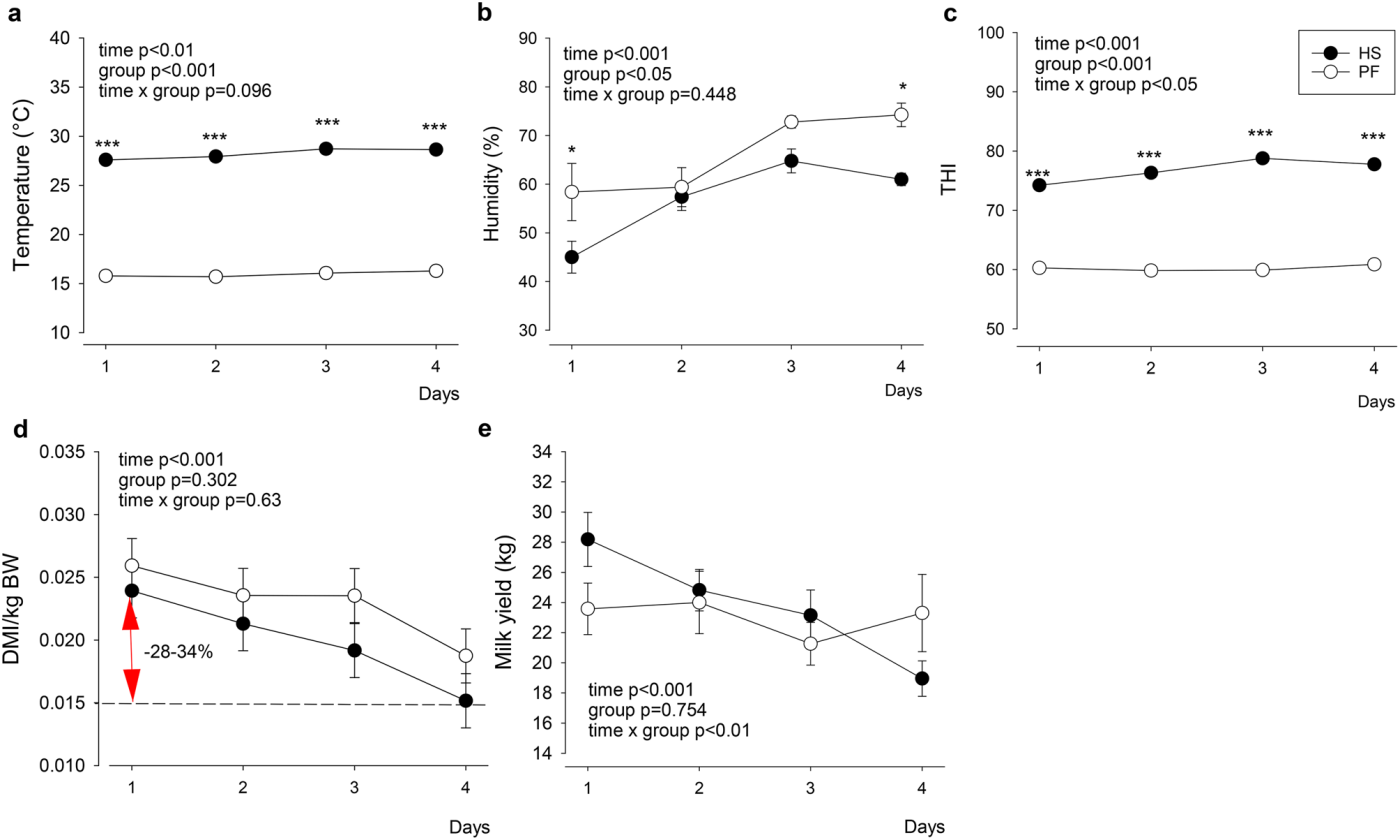
Temperature, humidity, feed intake, and milk yield during 4 days of heat stress (HS, full circles) or pair-fed (PF, open circles) conditions. (**a**) Ambient temperature, (**b**) humidity, (**c**) calculated temperature-humidity index (THI), (**d**) daily dry matter intake (DMI) per kg body weight (BW), and (**e**) daily milk yield. HS n = 5, PF n = 5. Data are given as LSM ± SE. * *P* < 0.05, *** *P* < 0.001. ANOVA (Tukey–Kramer). Graphs were created using Sigma plot (version 14.0; Systat Software, San Jose, CA, USA).

The ambient temperature and the humidity ranged between 27.4–29 °C, and 37–73%, respectively, resulting in a THI of 75–79 for the HS group (Fig. 1a–c). For the PF group kept at thermoneutral conditions, the temperature maintained between 14.7 and 16.4 °C and the humidity ranged between 43 and 79%, resulting in a THI of 60–61. Dry matter intake per kg body weight decreased by 28–34% after 4 days of challenge (time *P* < 0.001) in both groups (Fig. 1d). In addition, milk yield decreased by 26% in the HS group and by 7% in the PF group after 4 days of challenge (time × group *P* < 0.01; Fig. 1e). At the end of the experimental period, rectal temperature of HS or PF amounted to 40.2 and 38.4 °C, respectively (group *P* < 0.001; Fig. S1a). The respiration frequency increased from 77 to 95 breaths per min in HS but remained unaltered in PF animals (time x group *P* < 0.05; Fig. S1b), whilst the heart rate did not change over time or between groups (Fig. S1c).

### Metabolites, enzyme activities and amino acid composition in jejunal mucosa

The glucose concentration tended to be higher in HS than in PF cows (*P* = 0.1), whilst there was no difference in the mucosal lactate concentration (Table 1). The tissue aspartate aminotransferase (AST), glutamate dehydrogenase (GLDH) and lactate dehydrogenase (LDH) enzyme activities were not different among groups. Furthermore, individual, total, ketogenic, glucogenic, essential and non-essential amino acid concentrations did not differ between the mucosa of HS and PF cows (Table 2).

**Table 1:** Glucose and lactate concentrations and enzyme activities of AST, GLDH and LDH in jejunal mucosa of dairy cows exposed to heat stress (HS) or pair-feeding (PF) at thermoneutrality for 4 days.

Item	HS	PF	*P* value
Glucose (µmol/g protein)	31.0 ± 4.0	22.0 ± 4.0	0.1^#^
Lactate (µmol//g protein)	170.0 ± 11.0	164.0 ± 14.0	0.42
AST (U/g protein)	249.1 ± 21.4	251.4 ± 36.5	0.69
GLDH (U/g protein)	138.1 ± 25.4	140.6 ± 18.1	1.0
LDH (U/g protein)	184.8 ± 20.3	174.6 ± 52.1	0.42

HS n = 5, PF n = 5. Data are given as mean ± SEM (MWU test).

^#^0.06 < *P* < 0.1.

**Table 2:** Amino acid composition of the jejunal mucosa of dairy cows after 4 days of heat stress (HS) or pair-feeding (PF).

Metabolite (µmol/g protein)	Treatment		*P* value
	HS	PF	
Asp	31.5 ± 2.3	32.5 ± 5.2	0.69
Glu	101.3 ± 5.3	87.9 ± 10.7	0.42
Cys	10.9 ± 1.1	10.6 ± 1.7	1.00
α-AAA	3.2 ± 0.5	3.3 ± 0.6	1.00
Asn	12.8 ± 1.1	11.3 ± 1.6	0.42
Ser	35.8 ± 3.1	33.5 ± 4.9	0.55
Gln	21.6 ± 1.7	19.3 ± 2.7	0.69
His	6.6 ± 0.3	6.1 ± 1.0	0.15
Gly	157.7 ± 6.9	159.5 ± 11.1	1.00
Thr	21.3 ± 2.2	21.3 ± 3.0	0.69
Cit	3.9 ± 0.3	4.2 ± 0.6	0.55
β-Ala	3.5 ± 0.3	4.1 ± 0.5	0.31
Arg	28.9 ± 2.3	27.7 ± 4.5	0.69
Met-His	2.1 ± 0.4	1.4 ± 0.3	0.31
Ala	65.9 ± 3.6	63.6 ± 9.1	0.31
Tau	61.4 ± 7.0	61.0 ± 5.7	1.00
GABA	1.8 ± 0.2	1.7 ± 0.2	0.84
Tyr	29.2 ± 3.3	26.7 ± 4.3	0.84
α-ABA	1.1 ± 0.3	1.2 ± 0.3	0.69
Val	20.2 ± 1.4	20.4 ± 3.0	0.42
Met	12.7 ± 1.2	11.9 ± 1.6	0.55
Trp	4.8 ± 0.3	4.0 ± 0.3	0.22
Phe	28.1 ± 3.7	26.7 ± 4.6	0.69
Ile	16.5 ± 1.4	16.0 ± 2.3	0.55
Leu	42.6 ± 3.9	40.5 ± 6.2	0.55
Lys	50.0 ± 4.7	47.1 ± 7.5	0.55
Pro	10.2 ± 2.0	15.8 ± 3.7	0.31
∑ AA	709 ± 33	682 ± 85	0.42
Ketogenic AA	93 ± 8	88 ± 13	0.55
Glucogenic AA	617 ± 26	595 ± 71	0.42
Essential AA	204 ± 18	194 ± 29	0.55
Non-essential AA	506 ± 17	488 ± 56	0.42

HS n = 5, PF n = 5. Data are given as mean ± SEM (MWU test).

*AA* amino acid; *α-AAA* α-aminoadipic acid; *α-ABA* α-aminobutyric acid; *GABA* γ-aminobutyric acid.

### Differential proteomic analysis

**Figure 2:**
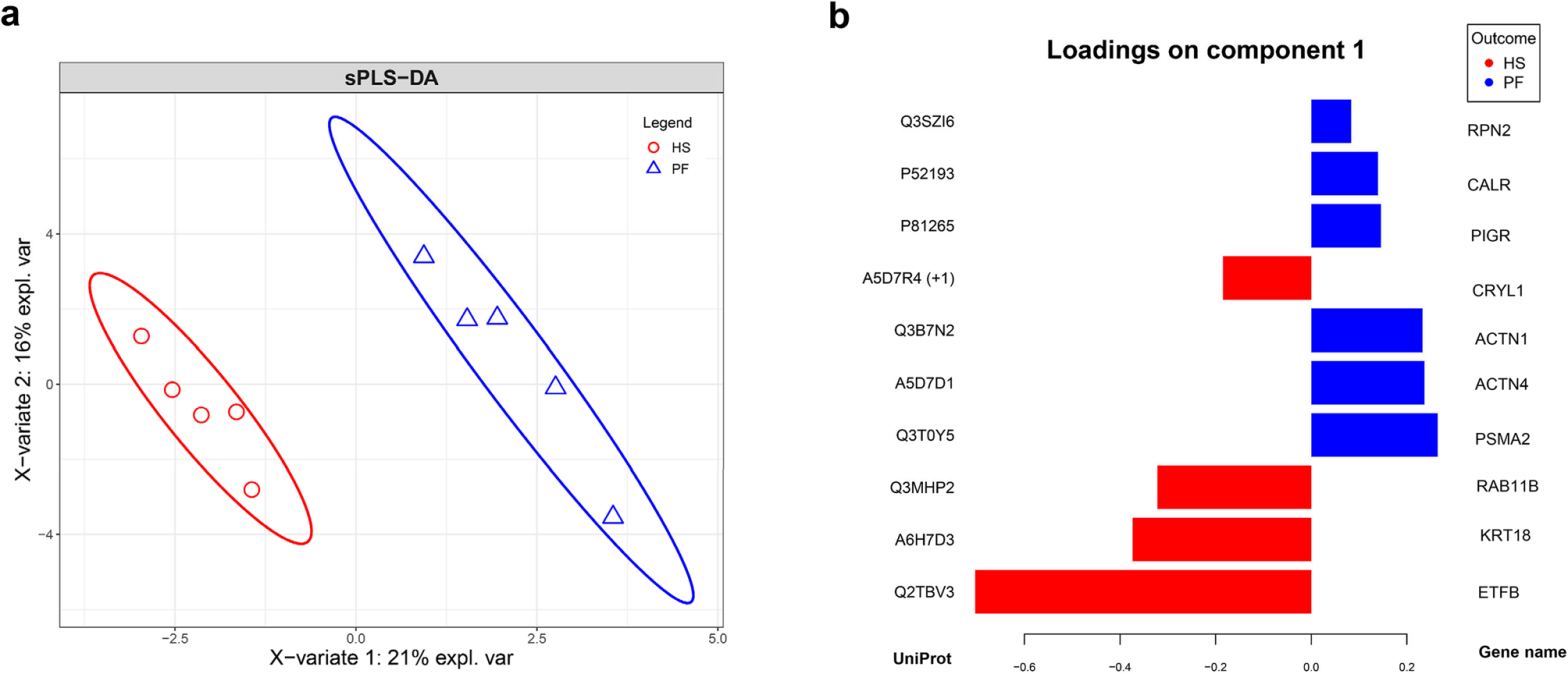
(**a**) Sparse Partial Least Squares-Discriminant Analysis (sPLS-DA) explains 21% of the variation 1 and 16% of the variation 2 (heat stressed (HS; red, open circles) and pair-fed (PF; blue, open triangles) dairy cows. (**b**) The loading on component one represent the main proteins behind the HS (red bars) and PF (blue bars) cluster. Graphs were created using the mixOmics package in R (version 6.1.1)^60^.

While a total of 5082 different proteins were identified in samples from all 10 cows (Table S1), only 899 proteins met our selection criteria of being present in at least 3 animals per group. Among these, 81 were tested significantly different between groups, with 59 upregulated and 22 downregulated mucosal proteins under HS compared to PF conditions (Table S2). The result of the Sparse Partial Least Squares-Discriminant Analysis (sPLS-DA) revealed clear discrimination between proteins of each group and explained 21% of the variation of component 1 and 16% of the variation of component 2 (Fig. 2a). Proteins contributing most to the cluster of the HS group were crystallin lambda 1 (CRYL1), Ras-related protein Rab-11B (RAB11B), kreatin 18 (KRT18), and electron transfer flavoprotein subunit beta (ETFB); and of the PF group dolichyl-diphosphooligosaccharide-protein glycosyltransferase subunit 2 (RPN2), calreticulin (CALR), polymeric immunoglobulin receptor (PIGR), alpha-actinin-1 (ACTN1), alpha-actinin-4 (ACTN4), proteasome subunit alpha type-2 (PSMA2) (Fig. 2b).

### Biological processes

**Figure 3:**
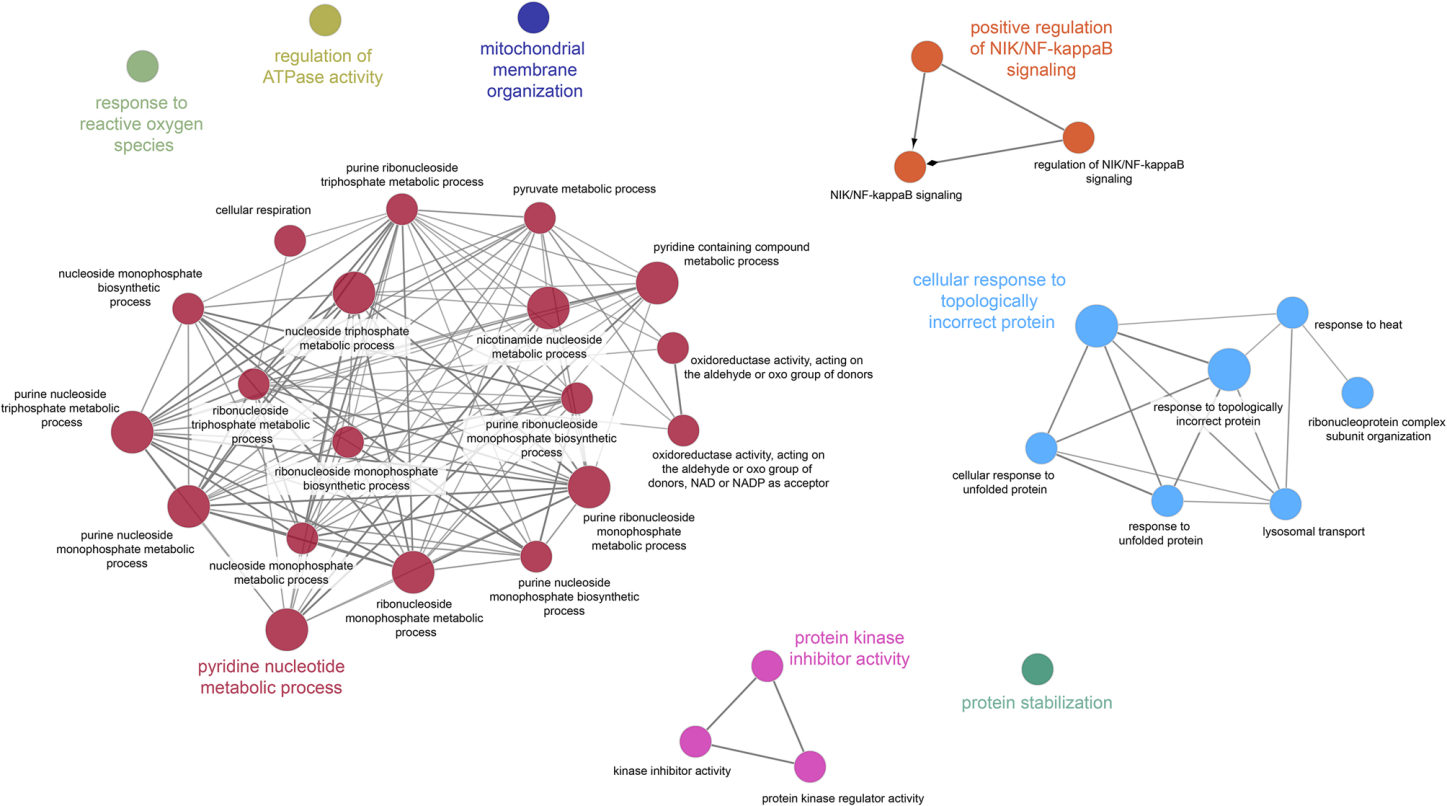
Functional classification of differentially (*P* < 0.05) expressed proteins in jejunal mucosa of dairy cows after 4 days of heat stress based on Gene ontology biological processes using ClueGO software^61^ (version 3.8.0; Cytoscape Software; https://cytoscape.org).

The ClueGO analysis identified 36 GO terms altered in the jejunal mucosa after heat stress (Table 3). These GO terms involve five major biological processes: (1) cellular responses to topologically incorrect protein folding, lysosomal transport and associated protein stabilization; (2) protein kinase inhibitor activity; (3) nucleoside metabolic processes including pyruvate metabolism; (4) regulation of ATPase activity, mitochondrial membrane organization, mitochondrial respiration and the response to reactive oxygen species; and (5) regulation of NIK/NF-κB signaling (Fig. 3, Table S2). More specifically, responses to incorrect protein folding and stabilization processes are characterized by upregulation of the heat shock protein 90 kDa protein alpha (HSP90AA1), heat shock cognate 71 kDa protein (HSPA8), heat shock 70 kDa protein 1A (HSPA1A) and 4 (HSPA4), heat shock 27 kDa protein 1 (HSPB1), the proteasome subunit alpha type-4 (PSMA4) and protein disulfide-isomerase A3 (PDIA3), but downregulation of the calcium-binding chaperones CANX protein (CANX) and CALR, the transitional endoplasmic reticulum ATPase (VCP), PSMA2, prolyl endopeptidase (PREP) and glucosidase II α subunit (GANAB) in HS cows (Tables 3 and S2). Among proteins assigned to the protein kinase inhibitor group were glutamine t-RNA ligase (QARS) and HSPB1, both higher abundant in HS than PF cows, while nucleophosmin (NPM1) assisting proteins in their transport to the nucleolus and involved in endoribonuclease activity was lower expressed in HS than PF animals.

**Table 3:** Leading terms of enriched Gene Ontology biological process (GO) with ClueGO classification program and differentially expressed jejununal mucosa proteins of heat stress vs pair-fed dairy cows.

GO:ID	GO term	Number of proteins involved	Protein names	*P* value
GO:0000302	Response to reactive oxygen species	3	CAT, RPS3, SOD2	4.54E−02
GO:0043462	Regulation of ATPase activity	3	ACTN1, ALDOB, HSPA1A	1.06E−02
GO:0050821	Protein stabilization	3	CALR, GAPDH, HSP90AA1	3.32E−02
GO:0007006	Mitochondrial membrane organization	3	HSP90AA1, HSPA4, SLC25A6	2.00E−02
GO:0019210	Kinase inhibitor activity	3	HSPB1, NPM1, QARS	1.81E−02
GO:1901224	Positive regulation of NIK/NF-kappaB signaling	3	ACTN4, CALR, RPS3	1.98E−02
GO:0009408	Response to heat	3	HSP90AA1, HSPA1A, VCP	4.14E−04
GO:0007041	Lysosomal transport	3	HSPA1A, HSPA8, VCP	4.14E−04
GO:0035967	Cellular response to topologically incorrect protein	5	CALR, CANX, HSPA1A, HSPA8, VCP	4.14E−04
GO:0071826	Ribonucleoprotein complex subunit organization	5	HSP90AA1, PRPF8, RPS19, SNRNP200, VCP	4.14E−04
GO:0016903	Oxidoreductase activity, acting on the aldehyde or oxo group of donors	3	ALDH18A1, DLD, GAPDH	4.87E−05
GO:0072524	Pyridine-containing compound metabolic process	5	ALDOB, GAPDH, NNT, TALDO1, VCP	4.87E−05
GO:0045333	Cellular respiration	3	CAT, DLD, VCP	4.87E−05
GO:0016620	Oxidoreductase activity, acting on the aldehyde or oxo group of donors, NAD or NADP as acceptor	3	ALDH18A1, DLD, GAPDH	4.87E−05
GO:0009141	Nucleoside triphosphate metabolic process	6	ADA, ALDOB, ATP5F1, DLD, GAPDH, VCP	4.87E−05
GO:0019362	Pyridine nucleotide metabolic process	5	ALDOB, GAPDH, NNT, TALDO1, VCP	4.87E−05
GO:0006090	Pyruvate metabolic process	3	ALDOB, DLD, GAPDH	4.87E−05
GO:0009124	Nucleoside monophosphate biosynthetic process	3	ADA, ATP5F1, VCP	4.87E−05
GO:0009199	Ribonucleoside triphosphate metabolic process	5	ALDOB, ATP5F1, DLD, GAPDH, VCP	4.87E−05
GO:0046496	Nicotinamide nucleotide metabolic process	5	ALDOB, GAPDH, NNT, TALDO1, VCP	4.87E−05
GO:0009156	Ribonucleoside monophosphate biosynthetic process	3	ADA, ATP5F1, VCP	4.87E−05
GO:0009167	Purine ribonucleoside monophosphate metabolic process	6	ADA, ALDOB, ATP5F1, DLD, GAPDH, VCP	4.87E−05
GO:0009205	Purine ribonucleoside triphosphate metabolic process	5	ALDOB, ATP5F1, DLD, GAPDH, VCP	4.87E−05
GO:0009168	Purine ribonucleoside monophosphate biosynthetic process	3	ADA, ATP5F1, VCP	4.87E−05

*P* value was calculated according to a hypergeometric test and corrected with Bonferroni stepdown in the ClueGo software.

The complexity of nucleoside metabolism includes pyruvate metabolism, the latter involving the glycolytic enzymes glyceraldehyde-3-phosphate dehydrogenase (GAPDH), fructose-bisphosphate aldolase B (ALDOB), aldehyde dehydrogenase 18 family member A1 (ALDH18A1), which were higher expressed in HS than PF cows. By contrast, the glycolytic enolase 1 (ENO1), a subunit of the pyruvate-dehydrogenase complex, dihydrolipoyl dehydrogenase (DLD), and adenosine deaminase (ADA) were lower abundant, whereas transaldolase (TALDO1), a key enzyme of the pentose phosphate pathway was higher abundant in HS than PF cows. For mitochondrial respiration and ATPase activity controlling oxidative phosphorylation, we found ATP synthase subunit alpha (ATP5A1), ATP synthase F(0) complex subunit B1 (ATP5F1), ADP/ATP translocase 3 (SLC25A6), NAD(P) transhydrogenase (NNT), and ETFB higher abundant in HS cows. Moreover, the mitochondrial superoxide dismutase 2 (SOD2) and peroxiredoxin-1 (PRDX1) detoxifying reactive oxygen species in the mitochondria were higher expressed, whilst cytosolic catalase was lower abundant in HS than PF animals. Among proteins positively regulating NIK/NF-κB signaling, we found α-actinin-4 (ACTN4) and CALR lower abundant, whereas 40S ribosomal protein S3 (RPS3) was higher abundant in HS compared to PF animals.

Besides the leading biological processes identified by ClueGO, further individual proteins were found differentially expressed. Proteins involved in cytosolic transportation and cellular stabilization, e.g. ACTN1 and ACTN4 were lower expressed in HS than PF animals. The proteins AP-2 complex subunit alpha-2 (AP2A2), RAB11B, Rho GDP-dissociation inhibitor 1 (ARHGDIA) controlling cellular trafficking and signaling were upregulated, but RPN2 downregulated in HS cows. Furthermore, the 40S ribosomal protein S19 (RPS19), 40S ribosomal protein S18 (RPS18) and 40S ribosomal protein S9 (RPS9) involved in ribonucleoprotein complex organization was higher expressed in HS than in PF cows. Further proteins related to fatty acid, protein and amino acid catabolism, namely cytosolic acetyl-coenzyme A acetyltransferase 2 (ACAT2), mitochondrial enoyl-CoA hydratase (ECHS1), and ornithine carbamoyl-transferase (OTC) were higher expressed in HS than PF animals, whereas aspartyl aminopeptidase (DNPEP) and alpha-aminoadipic semialdehyde dehydrogenase (ALDH7A1) were lower abundant in HS than PF cow. In addition, CRYL1 protein, converting L-gulonate to dehydro-L-gulonate in the uronate cycle functioning as an alternative glucose metabolic pathway, is upregulated under HS compared to PF conditions. Further proteins overexpressed during heat stress were short-chain dehydrogenase/ reductase 7 (DHRS7), isomerizing retinol, inter-alpha-trypsin inhibitor heavy chain H4 (ITIH4) and kininogen-1 (KNG1) controlling platelet degranulation and response to elevated platelet cytosolic calcium, hydroxysteroid (17-beta) dehydrogenase 11 (HSD17B11), and AKR1C4 protein (AKR1C4) regulating androgen catabolism and biotransformation of toxic compounds. The PIGR protein, related to the innate immune system, and alpha-1-acid glycoprotein (ORM) as an acute phase protein were lower expressed in HS than PF cows.

### Differential mRNA abundances

To examine if the adaptation processes identified on protein level can also be found at mRNA level. The mRNA expression of the heat shock 70 kDa protein A1 (*HSPA1A*) tended to be 1.7-fold higher expressed in HS than PF cows (*P* = 0.06), whilst heat shock protein 90 alpha family class B member 1 (*HSP90AB1*) was unaltered between the groups (Table S4). Heat stress had no effect on the mRNA abundance of enzymes involved in glutamine and lactate metabolism, glucose transport and energy metabolism, respiratory chain complexes and mitochondrial membrane proteins.

## Discussion

Cows exposed to high ambient temperatures in the present study showed significant signs of chronic heat stress such as higher rectal temperature and respiration rate, and lower feed intake with simultaneous reduction in milk yield over the course of the HS challenge. During heat stress, compensatory mechanisms protect vital organs, among others, by directing the visceral blood flow and thus endogenous heat towards the skin^23,24^. The resulting diminished blood flow in the small intestine induces local cellular hypoxia^25^, which consequently damages function of cells establishing the gut barrier^12^. Among the 5082 proteins identified, 4 tight-junction proteins (claudin3 and TJP1, TJP2 and TJP3) were included, but only in 3 to 4 out of 10 animals. Our filtering criteria excluded those proteins identified in less than 3 animals per group, and thus the expression analysis of tight-junction proteins was not be performed.

### Responses to incorrect protein folding, cellular stabilization processes, kinase inhibition and NF-κB signaling

Several heat shock protein members (HSP90AA1, HSPA8, HSPA1A, HSPB1. HSPA4), involved in the prevention and correction of protein misfolding, were overexpressed in the intestinal mucosa of HS cows suggesting an adaptational processes to maintain biological function of enterocytes under high ambient temperatures^12^. These results agree to a previous study reporting that HSPA1A, known as heat shock 70 kDa protein 1A, and heat shock 70 kDa protein 5 were higher abundant in the jejunum after 6 days of cycling heat stress periods^26^. In addition, high ambient temperatures induced cellular damage in porcine small intestine after 3 days of heat stress^16^, but the damage was repaired in the following days as indicated by increased cell proliferation and migration in crypts^27^. In addition, the expression of genes related to cell proliferation and migration changed from 6 to 10 days of HS in pigs^27^. In our study, downregulation of ACTN1 and ACTN4 involved in cell migration and stabilization was observed and indicates molecular destabilization of the cellular structure and barrier dysfunction along the heat-stressed mucosa in the intestine. The dysregulation of the tight junction barrier in the same animals was observed earlier in Koch et al*.*^14^. Our pathway enrichment analysis further showed that heat stress activates NF-κB signaling, a key inflammatory pathway, by lowering ACTN4, CALR and increasing RPS3 protein expression. This finding suggests an inflammatory response via the TLR4/NF-κB signaling cascade induced by invaded bacteria and pathogens^12,13,28^, further pointing to the disrupted gut barrier function of the intestinal mucosa of HS cows. In a previous study it has been shown that LPS-challenge simulating a higher pathogen loading under thermoneutral conditions in C57/BL6 mice increases the number CD177 positive neutrophils in the villi and activates the TLR4/IκB-α/ NF-κB signaling pathway at protein level in the jejunum^29^. However, we found the expression of NPM1, a phosphorylated protein with nucleolus transport function lower abundant, but QARS, catalyzing the aminoacylation of tRNA, higher abundant in HS than PF cows, suggesting that the biogenesis and transport of ribosomes rather than protein kinase activity are affected during heat stress.

### Glucose and energy metabolism

Although the majority of differentially expressed proteins were assigned to nucleoside metabolism by ClueGO, their primary role is rather in glucose and energy metabolism. We observed that heat stress increased ALDOB, GAPDH, CRYL1 and TALDO1 protein expression while *ALDOB* mRNA abundances were unaffected. The discrepancy between *ALDOB* mRNA and protein expression might be due to a high translation efficiency or because of the posttranscriptional modification of aldolase B protein^30^. The latter regulates the activity of aldolase and thus rates of glycolysis/gluconeogenesis, the pentose phosphate pathway, and fructose and mannose metabolism^31^. In addition to its function in glycolysis, GAPDH has important roles in DNA repair and replication, post transcriptional regulation, gene expression, and cell death^32,33^. The CRYL1 protein was first isolated from rabbit lenses but also has L-gulonate 3-dehydrogenase activity^34^. This enzyme is part of the uronate cycle which degrades glucose-6-phosphate via glucuronic acid towards xylose. Increased glucuronic acid may originate from the degradation of mucopolysaccharides of the gut mucus, thereby activating the uronate cycle. Thus, increased CRYL1 expression suggests mucus degradation yielding xylose which feeds into the pentose phosphate cycle. Furthermore, the TALDO1 protein plays a key role in the non-oxidative state of the pentose phosphate pathway by producing glycolytic intermediates, such as glyceraldehyde-3-phosphate and fructose-6-phosphate^35^. Hence, increased expression of CRYL1 and TALDO1 suggest that the jejunal mucosa adapts to heat stress by activating the uronate cycle and pentose phosphate pathway.

However, we found no differences in lactate metabolism as indicated by comparable lactate concentrations, *LDHA/B* and *SLC16A1* mRNA expression. In addition to this finding, heat stress downregulated the glycolytic enzyme ENO1^36^ and the metabolic energy regulator DLD in glucose, pyruvate metabolism and TCA^37^. These downregulations and the tendency for a higher glucose concentration in the jejunal mucosa support the idea of diminished glucose utilization by the jejunal mucosa. The tending higher tissue glucose level might also be supported by increased glucose absorption via the glucose transporters GLUT2 and SGLT1. We found comparable *SLC2A2* and *SLC5A1* expressions in HS and PF cows, suggesting that glucose absorption was not altered during heat stress. This conclusion seems to contrast earlier reports on impaired nutrient absorption after heat stress and hypoxia in rats^38^. However, the amount of glucose ruminants absorb from the small intestine is only small even under normal physiological conditions^39^. Another potential reason for the tending higher glucose levels in the jejunal mucosa after heat stress might be the upregulation of gluconeogenesis. In nutrient-limited states (e.g. fasting), glucose generation by intestinal gluconeogenesis (e.g. glutamine) accounts for 20% in humans^40^. However, gluconeogenic activity in the intestinal mucosa of dairy cows might not play a major role in glucose anabolism due to the low abundance of *G6PC* and *PEPCKM* mRNA in the jejunum compared to the liver, the major gluconeogenic organ in dairy cows^41^. Our results exclude that gluconeogenesis contributes to the higher glucose levels because the mRNA abundances of *G6PC*, *PEPCKM*, *PEPCKC*, and *PC* were not different between groups. To sum up, as glucose absorption and gluconeogenesis were not affected by heat stress, diminished glucose utilization is the major factor increasing mucosal glucose concentrations, likely in an attempt to provide more glucose as fuel for immune cells in the *Lamina propria* which in turn defend against invading toxins, bacterial compounds, and pathogens when the barrier function is compromised during heat stress^14^.

Our results from the proteome analysis further indicates that the higher ACAT2 and ECHS1 expressions in HS cows facilitate the production of acetyl-CoA from fatty acids, likely to replenish TCA cycle intermediates because of reduced glycolysis. However, the upregulation of ACAT2 could not be confirmed on mRNA level. Besides, we noted higher ALDH18A1 protein expression in HS cows, suggesting a greater conversion of glutamate to delta 1-pyrroline-5-carboxylate^42^. However, we did not detected differences in other key enzymes involved in glutamate catabolism, namely GLS, GLUD1, CPS1, AST, and GLDH. Similarly, the concentration of glutamate and other amino acids in the mucosa tissue was not different between groups, suggesting no preference of HS cows for utilizing certain amino acids. Nevertheless, we found reduced expression of DNPEP, an aminopeptidase regulating endocytic sorting and recycling in protein metabolism^43^ as well as increased expression of PSMA4, the α3 subunit of the core 20S proteasome^44^, indicating proteolytic degradation of specifically misfolded and/or incorrectly folded proteins after the HS challenge. As a consequence of an increased proteolysis, upregulation of amino acid degradation in the urea cycle is required. Indeed, we observed higher OTC protein expression in HS animals. Thus, we conclude increased proteolysis and amino acid degradation in the mucosa of HS cows, but activation of these pathways did not alter the tissue amino acid profile or utilization preference for a specific amino acid.

### Mitochondrial respiration, response to oxidative stress

Numerous proteins associated with the electron transport chain and oxidative phosphorylation were found upregulated after heat stress, including NNT, ETFB, ATP5A1, ATP5F1 and SLC25A6. By contrast, Cui et al. (2015) reported reduced protein abundances of NDUFA10, NDUFS3, NDUFS1, UQCR1, ATP5A1, and ATP5B in the jejunal mucosa of pigs undergoing a 3-week heat stress challenge compared with ad libitum fed animals at thermoneutrality^45^. This discrepancy can be explained by the fact that the reference group (pair-feeding vs. ad libitum) studied, as well as the duration of heat stress influence the magnitude and direction of protein expression. In the present study, upregulation of proteins involved in oxidative phosphorylation was accompanied by signs of reduced glycolysis but increased amino and fatty acid catabolism. However, we found no hints of altered TCA cycling and thus NADH production. Thus, it remains speculative if the higher expression of proteins related to oxidative phosphorylation occurred as response to higher NADH production, reduced mitochondrial uncoupling or a combination of both.

Moreover, our proteome data revealed lower ALDH7A1 protein expression in HS cows, and lower ALDH7A1 protein expression and enzyme activity reduces energy consumption during hypoxia and starvation by promoting cellular energy homeostasis in HeLa and HEK293 cells^46^. Again, our results reflects the reduced energy production and hypoxic episodes in the small intestine of dairy cows during the HS compared to PF challenge.

When assessed on the mRNA level, we found the expression of genes related to respiratory chain complexes (*mt-ND1, NDUFV2, SDHD, mt-CYTB, COX1, mt-COX2, mt-COX3, ATP5A1, ATP5B, mt-ATP6*) or uncoupling proteins (*UCP2*, *UCP3*) not increased after heat stress. Previous studies agree to our findings showing that the expression of the respiratory chain complexes in liver and pancreatic islets are not in parallel on the mRNA and protein levels^47,48^ due to different translation efficiencies and the regulation of activities by posttranscriptional modifications^49^.

High ambient temperatures and concomitantly occurring cellular hypoxia increases the production of reactive oxygen species (ROS) in the small intestine^25^. We found higher SOD2 and PRDX1 protein expression in HS cows supporting earlier findings on increased ROS levels during heat stress. Furthermore, alkaline phosphatase activity and *CAT* mRNA expression were found increased the mid-jejunum of the same HS cows as studied herein^14^, further arguing for the rise of oxidative defense in HS cows. However, the proteomics data of the present study revealed lower catalase protein expression, contrasting our earlier finding on a higher catalase mRNA abundance in HS compared to PF cows^14^. Thus, we can only speculate if the catalase enzyme is subjected to increased proteolysis, and if the increase in CAT mRNA abundance^14^ occurs as a counter-regulatory mechanism to meet reduced catalase activity in the mucosa of heat-stressed animals.

Taken together, our findings of numerous proteins differentially expressed between PF and HS cows suggests that the function of energy production and oxidative stress defense of the mitochondria is tremendously affected under heat stress conditions and this result adds to previous studies describing that high ambient temperatures induce morphological changes with swelling of the mitochondria, broken cristae, and low matrix density in the small intestine rodents^50^.

There are not very many studies investigating the impact of heat stress on other organs of dairy cows. Wang et al. (2017) reported that the heat shock proteins HSP90A, HSP90B, HSPA6 were upregulated in the liver of Holstein dairy cows in summer relative to spring season^51^. In an earlier study, we observed an upregulation of HSPH1 and HSPB1 in rumen papillae of HS compared to PF Holstein cows^52^, suggesting a common upregulation of HSPs in visceral organs under heat stress conditions. However, results obtained from a proteome analysis differed between hepatic and jejunal respiratory chain complexes. In liver, proteins of the electron transport system were downregulated^51^, whereas several proteins involved in the regulation of the ATPase activity and mitochondrial respiration were upregulated in jejunal mucosa. This discrepancy can be explained by the different experimental designs. While cows sampled in summer and spring were fed ad libitum in the Wang et al. study^51^, we compared HS and PF animals. Besides, the blood flow and thus the degree of hypoxia might be different between liver, jejunum and rumen papillae, suggesting that the metabolism of individual organs adapt differentially during heat stress.

However, a limitation of the present study is that we could not distinguish between different cell types in the jejunal mucosa, including enterocytes and immune cells. A previous study demonstrated that activated immune cells in the mucosa prefer glucose and glutamate utilization and these nutrients are less available to the enterocyte metabolism^17^. Nonetheless, this work demonstrates the importance of heat stress induced metabolic alterations and environmental adaptations in one of the largest organs of the body, the gut.

In conclusion, the present study provides evidence that heat stress in dairy cows causes cellular destabilization, incorrect protein folding, increased proteolytic degradation, reduced glycolysis, and activation of NF-κB signaling, uronate cycling, pentose phosphate pathway, fatty acid and amino acid catabolism, mitochondrial respiration, ATPase activity and the antioxidative defense system. These adaptive metabolic and immune response mechanisms can be interpreted as attempt to maintain the biological function in the small intestine of heat-stressed dairy cows.

However, in order to understand the regulatory processes in the whole gastrointestinal tract during heat stress, investigations of further distal parts of the digestive tract, e.g. the hindgut are necessary.

## Methods

### Animal selection and treatment

Ten German Holstein cows were randomly assigned to heat stress (HS, n = 5) or pair-fed (PF, n = 5) group. Pair-feeding served as a control to achieve isocaloric intake between groups^53^. All cows were in established 2nd lactation (HS: week 28 ± 8; PF: week 39 ± 16; *P* > 0.3). Animals were adapted to the climate chamber at thermoneutral conditions (15 °C) for six days and received a total mixed ration twice daily at 0700 h and 1500 h^8^. As described recently, five HS cows were subsequently exposed for four days to 28 °C with 52 ± 2% relative humidity (RH) resulting in a temperature-humidity index (THI) of 76 with ad libitum feeding^14^. Cows had free access to water, feed and water tempered to 28 °C. The reduction of daily ad libitum intake of HS cows was calculated as percentage of the mean daily feed intake to provide the same amount of feed energy to PF cows under thermoneutral conditions. Feed intake was recorded daily. The five PF cows were exposed for four days to 15 °C with 63 ± 1% RH and a THI of 60. Animals were sacrificed in the institutional slaughterhouse, and jejunum samples and jejunum mucosa scrapings were taken and frozen in liquid nitrogen and stored at − 80 °C until later analysis. All procedures were approved by the ethics committee of the State Government in Mecklenburg-West Pomerania, Germany (LALLF M-V/TSD/7221.3-1.1-074/12). All methods were carried out in accordance with the German animal welfare act and in compliance with the ARRIVE guidelines.

### Glucose, lactate, aspartate aminotransferase and glutamate dehydrogenase analysis

For the analyses of metabolites and enzyme activities, 30 mg of powdered mucosa tissue was homogenized in 300 µl lysis puffer containing 10 mM HEPES (Thermo Fisher Scientific, Schwerte, Germany), 1% (v/v) Tween20 (Carl Roth, Karlsruhe, Germany), 1 mM EDTA (GE Healthcare, Munich, Germany), 10 mM NaF (Thermo Fisher Scientific), 0.1% (v/v) Triton X-100 (GE Healthcare), 0.5% (v/v) DOC (Sigma-Aldrich), 0.1% (w/v) SDS (USB Corporation, Cleveland, OH, USA) with 0.5 cycles and 80% amplitude (20-times) Ultrasonic Processor UP50H (Hielscher Ultrasound Technology, Teltow, Germany)^54^. The homogenized extract was centrifuged at 3000×*g* for 20 min at 4 °C. The supernatant was used to measure glucose and lactate concentrations and aspartate aminotransferase (AST) and glutamate dehydrogenase (GLDH) activities photometrically (Abx Pentra 400; Horiba, Kyoto, Japan) using kits for glucose (no. A11A01667, Axon Lab, Reichenbach, Germany), lactate (no. A11A01721, Axon Lab), AST activity (no. A11A01629, Axon Lab) and GLDH activity (LT-GD 0010, Labor + Technik Eberhard Lehmann GmbH, Berlin, Germany). Protein concentrations of the extracts were measured using the Bradford kit (Thermo Fisher Scientific). Metabolites and enzyme activities were normalized to the protein concentration of the mucosa extract.

### Mucosal amino acid analysis

Mucosa extracts gained as aforementioned were diluted with ultra pure water (1:20), and free amino acids were analyzed by HPLC equipped with a fluorescence detector (Series 1260 Infinity II/ 1200, Agilent Technologies, Germany). The HPLC method was adapted from Krömer et al.^55^. Briefly, amino acids were separated after automated pre-column derivatization with ortho-phthalaldehyde/3-mercaptopropionic acid and 9-fluorenylmethoxycarbonyl chloride after reaction with 3-mercaptopropionic acid as reducing agent and iodoacetic acid to block sulfhydryl groups. Analyses were carried out at a flow rate of 0.8 ml/min within 45 min on a 250 × 4 mm Hyperclone ODS (C18) 120 Å column protected by a 4 × 3 mm C18 pre-column (Phenomenex, Aschaffenburg, Germany) using a gradient with 40 mM phosphate buffer (pH 7.8) and acetonitrile/methanol/water (v:v:v: 45:45:10) ranging from 6 to 100%. Amino acid concentrations were normalized to the protein concentration of each sample.

### Proteome and bioinformatic analysis

Mucosa tissue (30 mg) was homogenized in lysis buffer (pH 7.8) containing 50 mM Tris–HCl (Carl Roth), 1 mM EDTA (GE Healthcare), 10 mM NaF (Thermo Fisher Scientific), 1% (v/v), IGEPAL CA-630 (Sigma-Aldrich), 0.1% (v/v) Triton X100 (GE Healthcare), 0.5% (v/v) deoxycholic acid (DOC; Sigma-Aldrich), 0.1% SDS (USB Corporation) and Roche complete Protease Inhibitor Cocktail tablets (one tablet/10 ml buffer; Roche Diagnostics, Mannheim, Germany). The homogenized extract was centrifuged at 13,000 rpm for 20 min at 4 °C. The protein concentrations of the supernatant were measured using the Bradford kit (Thermo Fisher Scientific). Equal amounts of protein (25 µg) were separated on a 15% SDS-PAGE. The gel was stained with Coomassie brilliant blue (Serva Electrophoresis GmbH, Heidelberg, Germany) overnight and washed with A. dest. The gel was sliced in 8 horizonal lines yielding 8 slices per lane (8 slices per animal, 80 slices in total). Each slice was transferred into an 1.5 ml reaction tube and washed twice with 100 µl of a solution with 50% CH_3_OH and 50% 50 mM NH_4_HCO_3_ for 30 min, and once with with 100 µl of 75% CH_3_CN for 10 min. Samples were dried at 37 °C for 20 min and incubated with 4 µg/ml trypsin solution overnight at 37 °C. For extraction, gel slices were covered with 60 µl of 0.1% trifluoroacetic acid in 50% CH_3_CN and incubated under shaking for 30 min. The peptide-containing supernant was transferred into a clear glass vial and dried at 45 °C for 100 min a concentrator (Eppendorf, Hamburg, Germany). The dry peptides (non-reduced or alkylated) were resuspended in 10 µl of CH_3_CN/H_2_O/trifluoroacetic acid (50% vol /49.5% vol /0.5% vol). Peptides were separated and analyzed using a Protxeon easy nLCII-system (Thermo Scientific) coupled to a Thermo Scientific LTQ Orbitrap-XL mass spectometer. A 0.1 × 200 mm column with C18 Aeris Peptide (Phenomenex, Torrance, CA, USA) and a gradient of 0.5%/min (buffer A = 0.1% formic acid in water, Optima LC/MS; buffer B = 0.1% formic acid in 99.9% acetonitrile, Optima LC/MS; Fisher Scientific) at a flow rate of 0.3 ml/min was applied. For MS and MS/MS analysis, a full survey scan in the Orbitrap-XL with a mass range (m/z 300–2,000) and a Fourier transform (FT) resolution of 30,000 was followed by data-dependent fragmentation experiments of the 5 most intense ions. Data were acquired in a data-dependent “top 5” format, selecting the most abundant precursor ions from the FTMS scan (mass range 300–2,000 Da). The FTM scans were acquired with a resolution of 30,000 and a target value of 1.2 × 10^6^ in the Orbitrap analyzer. The ion-trap MS scans were acquired with uni mass resolution in the LTQ using 3,000 as target value, 2 as the default charge state, and a lower intensity threshold for MS2 of 3000 counts. The normalized collision energy in the collision-induced dissociation was 35 eV and a dynamic exclusion was defined by a list size of 500 with exclusion duration of 30 s. The spectra were acquired in the LTQ via collision-induced dissociation. The parameters for the dynamic exclusion list are as follows: repeat counts = 1, repeat duration = 30 s, exclusion list size = 500, and exclusion duration = 30. Data files were searched against the National Center for Biotechnology Information Bovine database (http://www.ncbi.nlm.nih.gov/) using Mascot Version 2.6.2 with the common contaminant `kreatin` specified. The Mascot search was carried out considering the following parameters: parent ion mass tolerance of 10 ppm, fragment ion mass tolerance of 0.80 Da, and Met oxidation (+ 15.99492 Da). Each Mascot search included the data from all 8 gel slices per lane and results loaded into Scaffold software (version 4.8.7., Proteome Software Inc., Portland, OR, USA). The Scaffold viewer was utilized to validate MS/MS based peptide and protein identifications. Peptide identifications were accepted with two peptides characterizing uniquely one protein with 95% probability to achieve a false discovery rate less than 0.1% by the Peptide Prophet algorithm^56^ with Scaffold delta-mass correction. Only proteins identified in at least 3 of 5 animals per group were considered for further analysis. For differential protein expression, the raw spectral counts (Table S1) were processed according to Yohannes et al. (2019)^57^ and Branson et al. (2016)^58^ utilizing the DESeq2 package^59^ of the Bioconductor repository in R (www.bioconductor.org). The raw spectral counts and the metadata were used to generate a DESeqDataSet object with DESeqSetFromMatrix. The DESeq function performed estimate size factors, estimate dispersions, and negative bionominal WALD test analysis. The proteome results included the log2 fold change and p-values (p ≤ 0.05) were extracted in Table 3. The Principal Component Analysis (PCA) and Sparse Partial Least Squares-Discriminant Analysis (sPLS-DA) were performed after normalization of the raw count data and analyzed by the mixOmics package in R^60^. Additionally, functional enrichment analysis of the results from DESeq2 was performed with the ClueGO software^61^ and applying database from the Kyoto Encyclopedia of Genes and Genomes (KEGG) database (update: August 2020). Results were visualized with the Cytoscape software version 3.8.0^62^. The ontology selection on the base of biological processes was performed by the right-side hypergeometric statistic test by utilizing the Bonferoni stepdown method.

### Reverse-transcriptase quantitative PCR (RT-qPCR)

Total RNA of jejunum mucosa was extracted from 20 mg tissue powder with innuPREP RNA mini kit (Analytik Jena, Berlin, Germany) and innuPREP DNase I (Analytik Jena). The RNA quality was determined with an Agilent 2100 Bioanalyzer (Agilent Technologies, Santa Clara, CA, USA). The RIN factors for mucosa were between 5.9 and 7.9 (mean: 7.25). First strand cDNA synthesis (750 ng RNA) was completed using SensiFAST cDNA synthesis kit (Bioline, Luckenwalde, Germany). Transcriptional expression was quantified by RT-qPCR. Primers were designed using Primer3 software^63^ (v0.4.0, Table S3). One PCR reaction contained 2 µl diluted cDNA (10 ng/µl), 3 µl H_2_O PCR grade, 0.5 µl of each primer (4 µM), and 6 µl 2 × Puffer SensiFAST SYBR No-ROX mix (Bioline) and was carried out in duplicates using the LightCycler 96 (Roche, Basel, Switzerland). Amplicons were sequenced on an ABI 3130 Genetic Analyzer (Life Technologies GmbH, Darmstadt, Germany). The obtained sequences were blasted using NCBI BLAST tool to confirm sequence identity. The efficiency of amplification was calculated using LinRegPCR software^64^ (v2014.4; Academic Medical Centre, Amsterdam, Netherlands), yielding efficiency values between 1.78 and 1.90 (Table S3). Data were quantified by qbase software (Biogazelle, Gent, Belgium) using *hypoxanthine phosphoribosyltransferase 1* (HPRT1) and *ribosomal protein L32* (RPL32) as reference genes (M-value 0.4; V-value 0.139).

### Statistics

Group effects were analyzed using the non-parametric Mann–Whitney U-test of the UNIVARIATE procedure of SAS (v9.4, SAS Institute Inc., Cary, NC, USA). Temperature, humidity, THI, DMI intake per kg body weight, milk yield, rectal temperature, respiration rate, and heart frequency were analyzed by repeated measurement ANOVA with the MIXED procedure of SAS software. The ANOVA models contained the fixed factors group (levels: PF, HS), time and the interaction group × time. Least-squares means (LSM) and their standard errors (SE) were computed for each fixed effect in the models, and all pairwise differences of LSmeans were tested by the Tukey–Kramer procedure. The SLICE statement of the MIXED procedure was used for performing partitioned analyses of the LSmeans for the interaction group × time. Results were considered as statistical significant at *P* < 0.05 and trends between 0.06 < *P* < 0.1.

## Supplementary Information

Supplementary Information 1.

Supplementary Information 2.

## Data Availability

The mass spectrometry proteomics data have been deposited to the ProteomeXchange Consortium via the PRIDE^65^ partner repository with the dataset identifier PXD025769 and 10.6019/PXD025769.
