# Supplementary material for: Jejunal mucosa proteomics unravel metabolic adaptive processes to mild chronic heat stress in dairy cows
Source: Sci Rep. 2021 Jun 14;11:12484. doi: 10.1038/s41598-021-92053-x (PMC8203643; doi:10.1038/s41598-021-92053-x)
Supplement: Supplementary file 1 — Supplementary Information 1. [file 41598_2021_92053_MOESM1_ESM.docx]

**Jejunal mucosa proteomics unravel metabolic adaptive processes to mild chronic heat stress in dairy cows**Franziska Koch, Dirk Albrecht, Solvig Görs, Björn Kuhla

**Supplementary Material**

**Table S2.** List of proteins up- (positive Log2fold change) and downregulated (negative Log2fold change) in heat-stressed compared to thermoneutrally pair-fed dairy cows (Wald test analysis of DEseq function).

| UniProt number | Protein name | Protein names | BaseMean | Log2fold change | *P* value |
| --- | --- | --- | --- | --- | --- |
| F1N757 | Uncharacterized protein |  | 10.36 | 2.24 | 0.003 |
| Q2KIS5 | Hydroxysteroid (17-beta) dehydrogenase 11 | HSD17B11 | 15.05 | 2.12 | 0.024 |
| E1BQ37 | Splicing factor proline and glutamine rich protein | SFPQ | 6.37 | 2.12 | 0.002 |
| Q3T052 | Inter-alpha-trypsin inhibitor heavy chain H4 | ITIH4 | 6.62 | 2.12 | 0.002 |
| P32007 | ADP/ATP translocase 3 | SLC25A6 | 12.68 | 2.10 | 0.026 |
| Q32PD5 | 40S ribosomal protein S19 | RPS19 | 3.96 | 2.06 | 0.027 |
| P11024 | NAD(P) transhydrogenase, mitochondrial | NNT | 10.45 | 1.89 | 0.011 |
| P02672 | Fibrinogen alpha chain | FGA | 29.42 | 1.90 | 0.000 |
| P13619 | ATP synthase F(0) complex subunit B1, mitochondrial | ATP5F1 | 6.10 | 1.71 | 0.020 |
| F1MLB8 | ATP synthase subunit alpha | ATP5A1 | 26.83 | 1.64 | 0.017 |
| E1BF59 | Plectin | PLEC | 31.12 | 1.61 | 0.031 |
| E1BNR0 | Apolipoprotein B | APOB | 19.93 | 1.58 | 0.026 |
| E1BH78 | Small nuclear ribonucleoprotein U5 subunit 200 | SNRNP200 | 3.60 | 1.58 | 0.035 |
| A0A0D8XF50 | Histone H3 |  | 7.24 | 1.57 | 0.015 |
| Q0VCK5 | AP-2 complex subunit alpha-2 | AP2A2 | 5.53 | 1.54 | 0.013 |
| Q3T169 | 40S ribosomal protein S3 | RPS3 | 10.24 | 1.53 | 0.049 |
| P38657 | Protein disulfide-isomerase A3 | PDIA3 | 23.09 | 1.48 | 0.049 |
| Q3MHH4 | Glutamine-tRNA ligase | QARS | 6.50 | 1.47 | 0.034 |
| F1MNV5 | Kininogen-1 | KNG1 | 5.70 | 1.44 | 0.012 |
| Q24K14 | Dehydrogenase/reductase (SDR family) member 7 | DHRS7 | 5.27 | 1.43 | 0.041 |
| P38409 | Guanine nucleotide-binding protein subunit alpha-11 | GNA11 | 5.27 | 1.42 | 0.010 |
| REFSEQ:XP_001252647 | similar to endopin 2B |  | 16.30 | 1.42 | 0.007 |
| P41976 | Superoxide dismutase [Mn], mitochondrial | SOD2 | 7.19 | 1.40 | 0.007 |
| Q17QG2 | Nuclear migration protein nudC | NUDC | 5.88 | 1.35 | 0.024 |
| E1BBY7 | Heat shock 70 kDa protein 4 | HSPA4 | 14.70 | 1.35 | 0.008 |
| A7Z025 | PRPF8 protein | PRPF8 | 6.90 | 1.34 | 0.025 |
| Q3SZ65 | Eukaryotic initiation factor 4A-II | EIF4A2 | 8.71 | 1.34 | 0.043 |
| P62993 | Growth factor receptor-bound protein 2 | GRB2 | 4.75 | 1.33 | 0.026 |
| Q3MHW4 | SQRDL protein | SQRDL | 6.34 | 1.29 | 0.023 |
| Q3ZCK9 | Proteasome subunit alpha type-4 | PSMA4 | 9.87 | 1.26 | 0.005 |
| Q3ZBG2 | AKR1C4 protein | AKR1C4 | 15.66 | 1.18 | 0.034 |
| Q17QI3 | Acetyl-Coenzyme A acetyltransferase 2 | ACAT2 | 6.63 | 1.17 | 0.016 |
| Q2KJH7 | Aldehyde dehydrogenase 18 family, member A1 | ALDH18A1 | 12.53 | 1.16 | 0.024 |
| Q27975 | Heat shock 70 kDa protein 1A | HSPA1A | 53.10 | 1.16 | 0.010 |
| F1MBF6 | similar to [ras-related protein Rab-6A isoform X3](https://www.uniprot.org/uniprot/A0A2Y9RK32) | RAB6A | 10.89 | 1.14 | 0.009 |
| A7MBI7 | Catechol O-methyltransferase | COMT | 11.78 | 1.13 | 0.011 |
| F1MTR1 | IQ motif containing GTPase activating protein 2 | IQGAP2 | 13.86 | 1.13 | 0.041 |
| E9RHW1 | Heat shock 27kDa protein 1 | HSPB1 | 24.17 | 1.13 | 0.018 |
| Q3T0R1 | 40S ribosomal protein S18 | RPS18 | 8.01 | 1.11 | 0.048 |
| Q08DJ3 | Far upstream element (FUSE) binding protein 1 | FUBP1 | 7.45 | 1.10 | 0.026 |
| P10096 | Glyceraldehyde-3-phosphate dehydrogenase | GAPDH | 34.72 | 1.09 | 0.025 |
| Q2TBV3 | Electron transfer flavoprotein subunit beta | ETFB | 17.39 | 1.05 | 0.001 |
| E1BLC0 | Agmatinase | AGMAT | 8.11 | 1.02 | 0.028 |
| G5E5C8 | Transaldolase | TALDO1 | 21.51 | 0.97 | 0.051 |
| Q58DM8 | Enoyl-CoA hydratase, mitochondrial | ECHS1 | 20.85 | 0.97 | 0.050 |
| P68509 | 14-3-3 protein eta | YWHAH | 21.98 | 0.92 | 0.024 |
| A5D7R4 | CRYL1 protein | CRYL1 | 13.61 | 0.87 | 0.021 |
| P19803 | Rho GDP-dissociation inhibitor 1 | ARHGDIA | 20.49 | 0.84 | 0.027 |
| P19120 | Heat shock cognate 71 kDa protein | HSPA8 | 97.79 | 0.82 | 0.016 |
| A6QLG5 | 40S ribosomal protein S9 | RPS9 | 19.02 | 0.81 | 0.054 |
| Q3T0S5 | Fructose-bisphosphate aldolase B | ALDOB | 67.33 | 0.80 | 0.041 |
| Q3MHP2 | Ras-related protein Rab-11B | RAB11B | 23.17 | 0.77 | 0.006 |
| F1MG05 | Elongation factor 1-gamma | EEF1G | 13.15 | 0.76 | 0.043 |
| F1MNG5 | Ornithine carbamoyltransferase, mitochondrial | OTC | 28.98 | 0.75 | 0.027 |
| Q76LV2 | Heat shock protein HSP 90-alpha | HSP90AA1 | 157.79 | 0.75 | 0.008 |
| P08779 | Keratin, type I cytoskeletal 16 | KRT16 | 126.16 | 0.71 | 0.032 |
| A6H7D3 | KRT18 protein (Fragment) | KRT18 | 71.71 | 0.70 | 0.003 |
| Q5E947 | Peroxiredoxin-1 | PRDX1 | 48.23 | 0.58 | 0.053 |
| P63103 | 14-3-3 protein zeta/delta | YWHAZ | 44.43 | 0.58 | 0.049 |
| A6QNJ8 | GANAB protein (Fragment) | GANAB | 54.26 | -0.54 | 0.042 |
| Q3ZBT1 | Transitional endoplasmic reticulum ATPase | VCP | 84.13 | -0.58 | 0.023 |
| P52193 | Calreticulin | CALR | 80.99 | -0.70 | 0.005 |
| A5D7D1 | Alpha-actinin-4 | ACTN4 | 110.12 | -0.75 | 0.003 |
| Q1RMK2 | IGHM protein |  | 27.14 | -0.76 | 0.053 |
| Q3T0Y5 | Proteasome subunit alpha type-2 | PSMA2 | 9.57 | -0.79 | 0.046 |
| Q3SZI6 | Dolichyl-diphosphooligosaccharide-protein glycosyltransferase subunit 2 | RPN2 | 17.04 | -0.86 | 0.014 |
| ENSEMBL:ENSBTAP00000024462 | 47 kDa protein |  | 43.06 | -0.90 | 0.043 |
| Q3B7N2 | Alpha-actinin-1 | ACTN1 | 93.74 | -0.91 | 0.001 |
| F1MQ37 | Myosin heavy chain 9 | MYH9 | 179.84 | -0.97 | 0.021 |
| Q3T160 | Nucleophosmin | NPM1 | 13.88 | -1.02 | 0.030 |
| Q3SZR3 | Alpha-1-acid glycoprotein | ORM | 17.98 | -1.09 | 0.011 |
| O02717 | Non-muscle myosin heavy chain (Fragment) |  | 77.49 | -1.10 | 0.013 |
| P00432 | Catalase | CAT | 65.42 | -1.18 | 0.041 |
| F1MB08 | Alpha-enolase | ENO1 | 58.30 | -1.31 | 0.021 |
| A7Z066 | Canx protein | CANX | 28.51 | -1.32 | 0.016 |
| F1N206 | Dihydrolipoyl dehydrogenase | DLD | 14.13 | -1.47 | 0.016 |
| A5D7C6 | Prolyl endopeptidase | PREP | 10.43 | -1.54 | 0.039 |
| P56658 | Adenosine deaminase | ADA | 87.13 | -1.62 | 0.017 |
| P81265 | Polymeric immunoglobulin receptor | PIGR | 24.69 | -1.65 | 0.000 |
| Q2HJH1 | Aspartyl aminopeptidase | DNPEP | 6.83 | -1.93 | 0.012 |
| E1BFG0 | Alpha-aminoadipic semialdehyde dehydrogenase | ALDH7A1 | 5.12 | -2.37 | 0.007 |

**Table S3.** Primer sequence.

| Gene |  | Primer sequence (5’ to 3’) | GenBank accession no. | Product size (bp) | Annealing (°C / s) | PCR efficiency | Ref. |
| --- | --- | --- | --- | --- | --- | --- | --- |
| *ACAT2* | forward | GCAGCCCAGTCGATAAGGAT | NM_001075549.1 | 155 | 60 | 1.88 |  |
|  | reverse | TCAGTGAGCCCGTCACAAAG |  |  |  |  |  |
| *ALDOB* | forward | GCTCTCCACCGTACTGTTCC | NM_001034485.2 | 120 | 60 | 1.85 |  |
|  | reverse | GGGCTTTGGTAGAGGGCAAA |  |  |  |  |  |
| *ATP5A1* | forward | AACAGGCGGTTGCTTATCGT | NM_174684.2 | 139 | 60 | 1.89 |  |
|  | reverse | CCACCACCAAAAGCATCGTT |  |  |  |  |  |
| *ATP5F1B* | forward | TGCTTTATTGGGCAGAATCC | NM_175796 | 152 | 60 | 1.86 | ^1^ |
|  | reverse | GATCCGTCAAGTCATCAGCA |  |  |  |  |  |
| *mt-ATP6* | forward | GCCGTAATTACAGGATTCCGC | NC_006853.1 | 76 | 60 | 1.92 |  |
|  | reverse | GTGGAGTGGGTGTTCCTTGT |  |  |  |  |  |
| *mt-COX1* | forward | TCGCTCCCTGTATTAGCAGC | NC_006853.1 | 138 | 60 | 1.86 |  |
|  | reverse | GACTTCGGGGTGTCCAAAGA |  |  |  |  |  |
| *mt-COX2* | forward | TCACTTTCATGACCACACGC | NC_006853.1 | 148 | 60 | 1.86 |  |
|  | reverse | GCGGGCAGAATGGTTCAGAT |  |  |  |  |  |
| *mt-COX3* | forward | AAGTCCCACTGCTCAACACC | NC_006853.1 | 105 | 60 | 1.90 |  |
|  | reverse | GTTGAGCCGTAAACTCCGTC |  |  |  |  |  |
| *CPS1* | forward | TAACAGGATTGGCTGCTGGT | NM_001192258.1 | 70 | 60 | 1.88 |  |
|  | reverse | ACAGGTTGATTCTGCCCTCTG |  |  |  |  |  |
| *mt-CYTB* | forward | CCTCACAGGCCTATTCCTAGC | NC_006853.1 | 130 | 60 | 1.89 |  |
|  | reverse | TGAAGCTCCGTTTGCGTGTA |  |  |  |  |  |
| *GLS* | forward | TTCTCCAACAACGAGGGCAA | NM_001077964.2 | 182 | 60 | 1.86 |  |
|  | reverse | AACCTGGGATCAGACGTTCG |  |  |  |  |  |
| *GLUD1* | forward | GCAGTGGTTGATGTGCCATTT | NM_182652.2 | 107 | 60 | 1.88 |  |
|  | reverse | TCCATGGTGAACCTCCTTGTG |  |  |  |  |  |
| *G6PC* | forward | AACTCCTCTGGGTAGCTGTGA | NM_001076124.2 | 139 | 60 | 1.88 |  |
|  | reverse | GGGAACTGCTTTATCAGCGG |  |  |  |  |  |
| *G6PD* | forward | CCTCTTCTACCTGGCCTTGC | NM_001244135.2 | 106 | 60 | 1.89 |  |
|  | reverse | CTTCTCCACGATGATGCGGT |  |  |  |  |  |
| *HK1* | forward | CACCGAACTGAAGGATGACCA | NM_001012668.2 | 154 | 60 | 1.89 |  |
|  | reverse | CTTGACTGTGGCTGTCGGAT |  |  |  |  |  |
| *HPTT1* | forward | TACTGCTACTGTGTGCTTAGG | NM_001034035.2 | 111 | 60 | 1.87 | ^2^ |
|  | reverse | CTACTGAAACACTGGCGGGAC |  |  |  |  |  |
| *HSPA1A* | forward | AGTCGTACGCCTTCAACATGA | NM_203322.3 | 79 | 60 | 1.90 |  |
|  | reverse | TTCTTCTTGTCCGCCTCGCT |  |  |  |  |  |
| *HSP90AB1* | forward | GACTGGGAAGATCACTTGGCA | NM_001079637.1 | 98 | 60 | 1.90 |  |
|  | reverse | AGGTCAAAAGGAGCCCGAC |  |  |  |  |  |
| *LDHA* | forward | TGGGGAGCATGGTGACTCTA | NM_174099.2 | 187 | 60 | 1.86 | ^1^ |
|  | reverse | AATGGCCCAGGATGTGTAGC |  |  |  |  |  |
| *LDHB* | forward | ACTTGGCATTCATCCCAGCA | NM_174100 | 90 | 60 | 1.88 | ^1^ |
|  | reverse | GCCACATTCACTCCACTCCA |  |  |  |  |  |
| *mt-ND1* | forward | ACGCACTAATCGGAGCCCTA | NC_006853.1 | 157 | 60 | 1.84 |  |
|  | reverse | CCATGCTGGGAGGATTAACCA |  |  |  |  |  |
| *NDUFV2* | forward | CACCTTGCATGCTCCGAAAC | NM_174565.3 | 130 | 60 | 1.87 |  |
|  | reverse | ACACAGGCCCCTAAACATTCA |  |  |  |  |  |
| *NNT* | forward | GCAGCTACTATCACGCCCTT | XM_024981195.1 | 90 | 60 | 1.86 |  |
|  | reverse | CGCGATGCCCAAACCTAGTA |  |  |  |  |  |
| *PC* | forward | ACACCAACTACCCCGACAATG | NM_177946.4 | 353 | 60 | 1.80 | ^3^ |
|  | reverse | CAGCGGGAGGTCAGGGAAG |  |  |  |  |  |
| *PDHA2* | forward | ATGTGTTCGGGAGGCAACT | NM_001077071.2 | 75 | 60 | 1.90 |  |
|  | reverse | AGCAGCTCCATCAGTATGGGT |  |  |  |  |  |
| *PCK1* | forward | ATGACAACTGCTGGTTGGCT | NM_174737.2 | 367 | 60 | 1.85 | ^4^ |
|  | reverse | TGGAGGCACTTGACGAACTC |  |  |  |  |  |
| *PCK2* | forward | GCCGTAGACCCAAAGGAGTC | NM_001205594.1 | 181 | 60 | 1.86 | ^4^ |
|  | reverse | TCAAGGTAGCGCCCAAAGTT |  |  |  |  |  |
| *PFKL* | forward | GCTACCTGGCTACTGTGACTG | NM_001080244.2 | 158 | 60 | 1.87 |  |
|  | reverse | CTTCTCGTTCCGGAGTACCAG |  |  |  |  |  |
| *PKLR* | forward | AGAACCACGAGGGCGTAAAG | NM_001076176.1 | 225 | 60 | 1.84 |  |
|  | reverse | CACTTGTCTCTGCCCGAGTT |  |  |  |  |  |
| *RPL32* | forward | AGGGTGCGCAGAAGATTCAA | NM_001034783.2 | 188 | 60 | 1.88 |  |
|  | reverse | TTGGAGGAGACGTTGTGAGC |  |  |  |  |  |
| *SDHD* | forward | AATCCGTGTTCTGCGATGGA | NM_174179.2 | 77 | 60 | 1.89 |  |
|  | reverse | ACAACTTGTCCAATGCCCCA |  |  |  |  |  |
| *SLG2A2* | forward | TGGAGCTCTCTTGATGGGGT | NM_001103222.1 | 145 | 60 | 1.86 |  |
|  | reverse | CCTGAGTGTGGTTGGAGCAA |  |  |  |  |  |
| *SLG5A1* | forward | TGTCCCTGGTGCTGTACATTT | NM_174606.2 | 130 | 60 | 1.87 |  |
|  | reverse | TAAAGGGCGGTGATTGCCA |  |  |  |  |  |
| *SLG16A1* | forward | TTAATGCCACCACCAGTGAA | NM_174606.2 | 164 | 60 | 1.88 |  |
|  | reverse | GAAGGAAGCAGCAATCAAGC |  |  |  |  |  |
| *UCP2* | forward | ACATCTCCCAATGTCGCTCG | NM_001033611.2 | 121 | 60 | 1.87 |  |
|  | reverse | TAAAGTGGCAAGGGAGGTCG |  |  |  |  |  |
| *UCP3* | forward | ACGTGGTGAAGATCCGCTTT | NM_174210.1 | 100 | 60 | 1.78 |  |
|  | reverse | TCTCTGGCGATGGTCCTGTA |  |  |  |  |  |
| *VDAC1* | forward | ACACCGAGACCACCAAAGTG | NM_174485.4 | 119 | 60 | 1.88 |  |
|  | reverse | CACAGTAATCTCCGTGCCCA |  |  |  |  |  |

**Table S4.** Relative mRNA abundances of genes involved in heat shock response, glutamine, glucose and lactate metabolism, and mitochondrial function in the jejunal mucosa of dairy cows after 4 days of heat stress (HS) or pair-feeding (PF). HS n=5, PF n=5. Data are given as mean ± SEM (MWU test).

| Pathway | Gene | HS | PF |  |  | *P* value |
| --- | --- | --- | --- | --- | --- | --- |
| Heat shock response | *HSP90AB1* | 1.17 ± 0.19 | 0.91 ± 0.20 |  |  | 0.42 |
|  | *HSPA1A* | 1.36 ± 0.19 | 0.82 ± 0.23 |  |  | 0.06# |
| Glutamine metabolism | *GLS* | 1.06 ± 0.04 | 0.96 ± 0.08 |  |  | 0.69 |
|  | *GLUD1* | 1.37 ± 0.06 | 0.96 ± 0.14 |  |  | 0.84 |
|  | *CPS1* | 0.95 ± 0.14 | 1.1 ± 0.10 |  |  | 0.42 |
| Glucose transporter | *SLC2A2* | 1.37 ± 0.29 | 0.96 ± 0.23 |  |  | 0.55 |
|  | *SGLT1(SLC5A1)* | 1.80 ± 0.78 | 0.84 ± 0.14 |  |  | 0.42 |
| Glucose & energy | *HK1* | 1.10 ± 0.07 | 0.97 ± 0.04 |  |  | 0.22 |
| metabolism | *PFKL* | 1.01 ± 0.22 | 1.13 ± 0.11 |  |  | 0.69 |
|  | *PKLR* | 1.39 ± 0.61 | 1.00 ± 0.28 |  |  | 0.84 |
|  | *G6PC* | 1.62 ± 0.37 | 0.85 ± 0.25 |  |  | 0.22 |
|  | *G6PD* | 1.04 ± 0.17 | 1.04 ± 0.07 |  |  | 0.69 |
|  | *PCK1* | 2.49 ± 1.46 | 0.82 ± 0.19 |  |  | 0.31 |
|  | *PCK2* | 1.12 ± 0.47 | 1.21 ± 0.27 |  |  | 0.55 |
|  | *PC* | 1.08 ± 0.21 | 0.96 ± 0.15 |  |  | 0.69 |
|  | *PDHA2* | 1.10 ± 0.21 | 0.96 ± 0.08 |  |  | 0.55 |
|  | *ALDOB* | 1.51 ± 0.38 | 0.97 ± 0.22 |  |  | 0.54 |
|  | *ACAT2* | 1.23 ± 0.21 | 0.95 ± 0.22 |  |  | 0.15 |
| Lactate metabolism | *SLC16A1 (MCT1)* | 1.10 ± 0.09 | 0.95 ± 0.09 |  |  | 0.22 |
|  | *LDHA* | 1.31 ± 0.48 | 0.89 ± 0.06 |  |  | 0.84 |
|  | *LDHB* | 0.98 ± 0.18 | 1.10 ± 0.11 |  |  | 0.55 |
| Respiratory chain complex | *mt-ND1* | 1.28 ± 0.28 | 0.90 ± 0.12 |  |  | 0.42 |
|  | *NDUFV2* | 0.97 ± 0.11 | 1.06 ± 0.10 |  |  | 0.42 |
|  | *SDHD* | 1.10 ± 0.13 | 0.97 ± 0.07 |  |  | 0.55 |
|  | *mt-CYTB* | 1.09 ± 0.20 | 0.88 ± 0.13 |  |  | 0.55 |
|  | *mt-COX1* | 1.18 ± 0.32 | 1.01 ± 0.15 |  |  | 1.0 |
|  | *mt-COX2* | 1.37 ± 0.30 | 0.86 ± 0.12 |  |  | 0.31 |
|  | *mt-COX3* | 1.23 ± 0.29 | 0.94 ± 0.09 |  |  | 0.84 |
|  | *ATP5A1* | 1.22 ± 0.21 | 0.95 ± 0.22 |  |  | 0.15 |
|  | *ATP5F1B* | 0.98 ± 0.19 | 1.09 ± 0.11 |  |  | 0.55 |
|  | *mt-ATP6* | 1.21 ± 0.26 | 0.93 ± 0.11 |  |  | 0.69 |
| Mitochondria related gene | *NNT* | 0.94 ± 0.15 | 1.08 ± 0.10 |  |  | 0.84 |
| Mitochondrial membrane | *UC2* | 1.02 ± 0.12 | 1.04 ± 0.14 |  |  | 1.0 |
|  | *UCP3* | 0.84 ± 0.16 | 0.52 ± 0.12 |  |  | 0.22 |
|  | *VDAC1* | 1.25 ± 0.30 | 0.91 ± 0.11 |  |  | 0.42 |

# 0.06 < *P <* 0.1

**Figure S1.** (**a**) Rectal temperature, (**b**) respiration frequency and (**c**) heart rate measured in heat stress (HS, full circles) or pair-fed (PF, open circles) cows during 4 days of treatment. HS n=5, PF n=5. Data are given as LSM ± SE. * *P* < 0.05, *** *P* < 0.001. ANOVA (Tukey-Kramer). Graphs were created using Sigma plot (version 14.0; Systat Software, San Jose, CA, USA).

**References**

1 Koch, F., Lamp, O., Eslamizad, M., Weitzel, J. & Kuhla, B. Metabolic Response to Heat Stress in Late-Pregnant and Early Lactation Dairy Cows: Implications to Liver-Muscle Crosstalk. *PloS one* **11**, e0160912, doi:10.1371/journal.pone.0160912 (2016).

2 Koch, F. *et al.* Heat stress directly impairs gut integrity and recruits distinct immune cell populations into the bovine intestine. *Proceedings of the National Academy of Sciences of the United States of America*, doi:10.1073/pnas.1820130116 (2019).

3 Hammon, H. M. *et al.* Performance and metabolic and endocrine changes with emphasis on glucose metabolism in high-yielding dairy cows with high and low fat content in liver after calving. *Journal of dairy science* **92**, 1554-1566, doi:10.3168/jds.2008-1634 (2009).

4 Gruse, J. *et al.* The Effects of Oral Quercetin Supplementation on Splanchnic Glucose Metabolism in 1-Week-Old Calves Depend on Diet after Birth. *The Journal of nutrition* **145**, 2486-2495, doi:10.3945/jn.115.218271 (2015).
